# Morphological characteristics of CT blend sign predict hematoma expansion and outcomes in intracerebral hemorrhage in elderly patients

**DOI:** 10.3389/fmed.2024.1442724

**Published:** 2024-10-01

**Authors:** Qian Wu, Na Chen, Yunxu Ren, Siying Ren, Fei Ye, Xu Zhao, Guofeng Wu, Likun Wang

**Affiliations:** The Affiliated Hospital of Guizhou Medical University, Guiyang, China

**Keywords:** elderly, intracerebral hemorrhage, blend sign, hematoma expansion, poor outcomes

## Abstract

**Background and purpose:**

The underlying basis of the blend sign on brain computed tomography (CT) in patients with intracerebral hemorrhage (ICH) is unclear. Few studies have examined the morphological alterations in the CT blend sign in ICH. Therefore, we assessed changes in the CT blend sign and their association with hematoma expansion (HE) and adverse outcomes in ICH patients.

**Methods:**

We recorded the clinical and radiographic parameters of patients with ICH and blend sign on brain CT. The patients were categorized into two groups, with changes in the relatively hypoattenuating region of the blend sign (CHB group) and with no changes in the relatively hypoattenuating region of the blend sign (NHB groups). We performed univariate and multivariate logistic regression analyses to examine the correlations between CHB and HE and poor outcomes. Furthermore, receiver operating characteristic curve analysis was used to confirm the predictive power of CHB.

**Results:**

In total, 183 patients were included in the study, of whom 74 (40.4%) demonstrated changes in the hypoattenuating region of the blend sign, whereas 109 (59.6%) did not. Compared with the NHB group, patients in the CHB group exhibited significantly higher levels of HE and adverse outcomes. After adjustment for confounding factors, CHB was independently associated with HE (odds ratio, 19.401 [95% CI, 7.217–52.163]; *p* < 0.001) and poor 3-month outcomes (odds ratio, 2.638 [95% CI, 1.391–5.003]; *p* = 0.003) in the multivariate analysis. The sensitivity, specificity, positive predictive value, and negative predictive value of CHB for predicting HE were 0.877, 0.768, 0.791, and 0.862, respectively, whereas these values for predicting poor outcomes were 0.789, 0.641, 0.688, and 0.752, respectively.

**Conclusion:**

Changes of a hypoattenuating region within the blend sign have good predictive accuracy for HE and short-term adverse outcomes in elderly patients with ICH.

**Clinical trial registration:**

ClinicalTrials.gov, NCT05548530.

## Introduction

Intracerebral hemorrhage (ICH), which accounts for 10–15% of all strokes, represents a critical healthcare challenge, due to its severity and associated mortality (1). Numerous clinical trials have indicated that medical and surgical interventions for ICH are associated with limited improvement (2, 3). Although there is currently no definitive treatment available for ICH, several studies suggest that hematoma expansion (HE) plays a pivotal role in predicting unfavorable prognosis and early functional deterioration (4). Certain radiological markers have been identified that predict HE and adverse outcomes (5, 6).

The current consensus is that the hypoattenuating area within the blend sign is caused by the fresh and unclotted active bleeding; however, the specific underlying cause is unclear (7). Our previous studies have demonstrated that the CT Blend Sign is composed of two parts of blood with different age in rabbits ICH model (8). Furthermore, previous studies have examined the associations of blend sign with HE and outcomes of ICH (9, 10). Nevertheless, few studies have investigated the associations between various characteristics of the blend sign and outcomes in individuals with ICH.

This study explored the associations of various characteristics of the blend sign at the initial and first follow-up computed tomography (CT) with hematoma volume and location. We hypothesized that the morphology of the blend sign changes over time, suggesting that a comprehensive analysis of sequential CT images can more accurately depict the morphological variations of the blend sign. The hypodense area of the blend sign may indicate the architecture of the cerebrospinal fluid circulation, with fluctuations in this region potentially regarding as indicators of adverse outcomes in this patient population, such as rebleeding and a poor prognosis.

## Methods

### Study design and participants

We retrospectively analyzed the medical records of elderly patients treated for ICH between January 1, 2018, and December 1, 2023. The inclusion criteria were as follows: (1) Presence of spontaneous ICH and blend sign. The blend sign was identified based on a distinct boundary visible between the hypoattenuating area and the adjacent hyperattenuating region; (2) age 60 years or above; (3) both the initial CT scan and the follow-up CT scan conducted within 24 h of admission. The exclusion criteria were as follows: (1) Presence of traumatic ICH, arteriovenous malformations, and aneurysms, or use of anticoagulation or antiplatelet therapy; (2)incomplete CT scans, or initial CT scan conducted after 24 h of admission; (3)insufficient clinical data (11).

### Clinical assessment and neuroimaging

Two experienced neurologists, with 7 and 10 years of expertise, collaboratively assessed the hematoma location, blend sign, hematoma growth, National Institutes of Health Stroke Scale (NIHSS) score, and Glasgow Coma Scale (GCS) score of all patients. The blend sign was identified based on a distinct boundary visible between the hypoattenuating region and the adjacent hyperattenuating area; a density difference exceeding 18 Hounsfield units (HUs); and the lack of a hypodense region of hematoma encased by the hyperdense region (7).

### Outcomes

Hematoma volume was estimated using the ABC/2 formula, with significant expansion defined as an absolute volume increase greater than 6 mL or an increase exceeding 33% (12). In-person interviews conducted by trained staff or phone calls were used to determine the 90-day modified Rankin Scale (mRS) scores. The mRS scores were used to assess the outcomes at 90 days after ICH, with outcomes categorized as favorable (mRS scores: 0–3) or unfavorable (mRS scores: 4–6), in accordance with our previous research (13). Complication rates were recorded throughout the hospital stay to facilitate group comparisons.

### Groups

**Figure 1:**
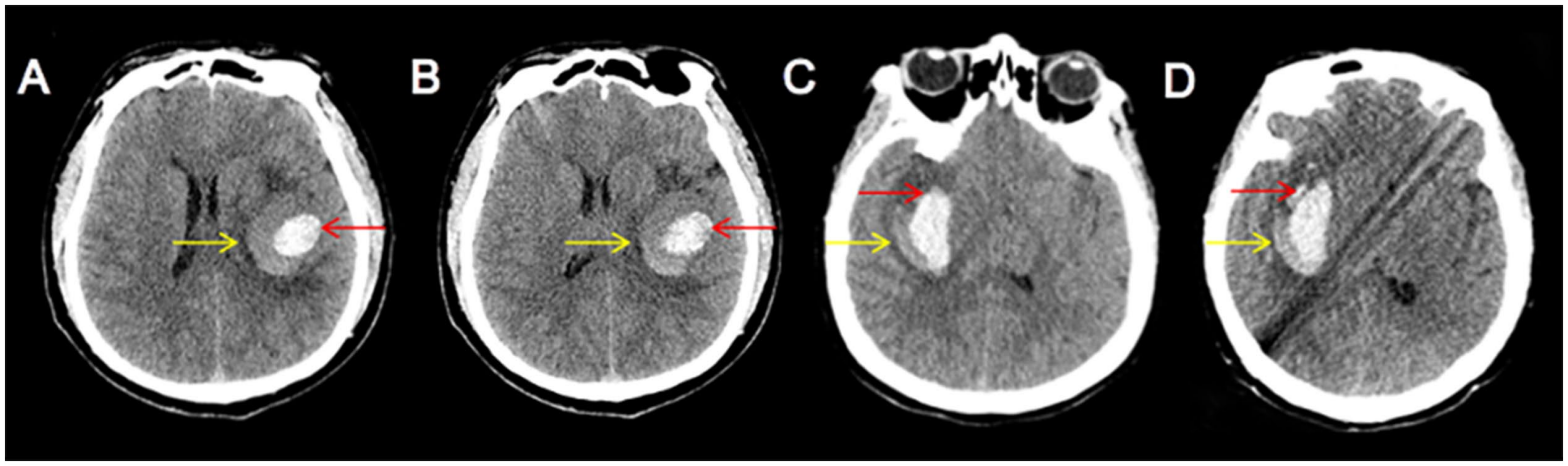
The hypodense area of the blend sign was unchanged. Craniocerebral CT images of two patients with ICH at admission **(A,C)** and follow-up **(B,D)**. Red arrow indicates the hyperdense area of the blend sign, and yellow arrow indicates the hypodense area of the blend sign. The two patients were categorized into the NHB group.

**Figure 2:**
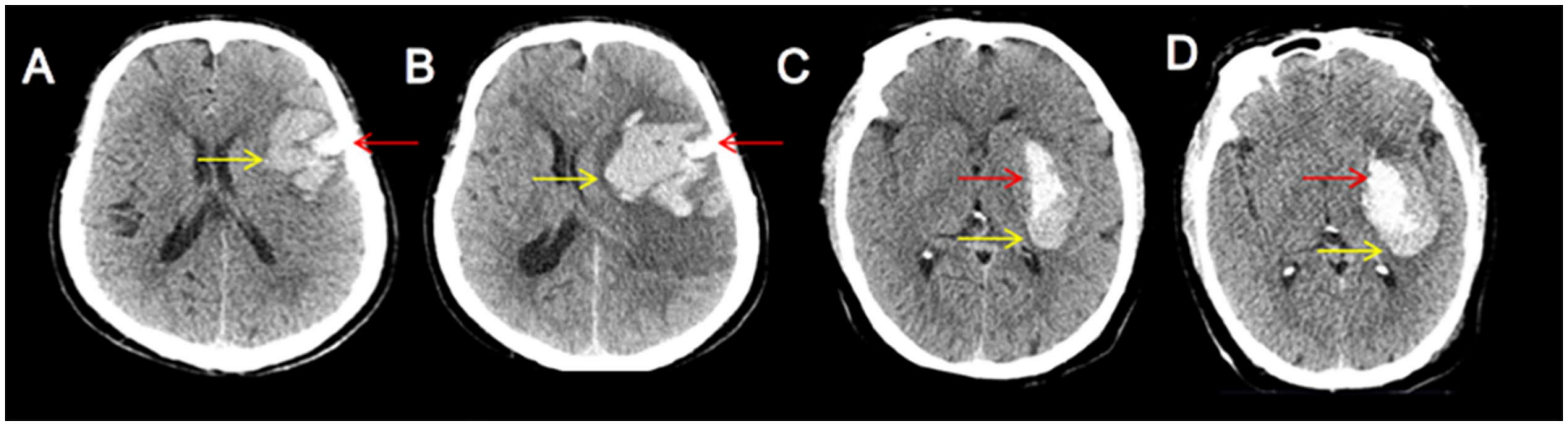
Two patients with increased hypodensity in the blend sign. Craniocerebral CT images of two patients with ICH at admission **(A,C)** and follow-up **(B,D)**. Follow-up CT showed increased hypodensity in the blend sign (yellow arrow). These two patients were categorized into the CHB group.

**Figure 3:**
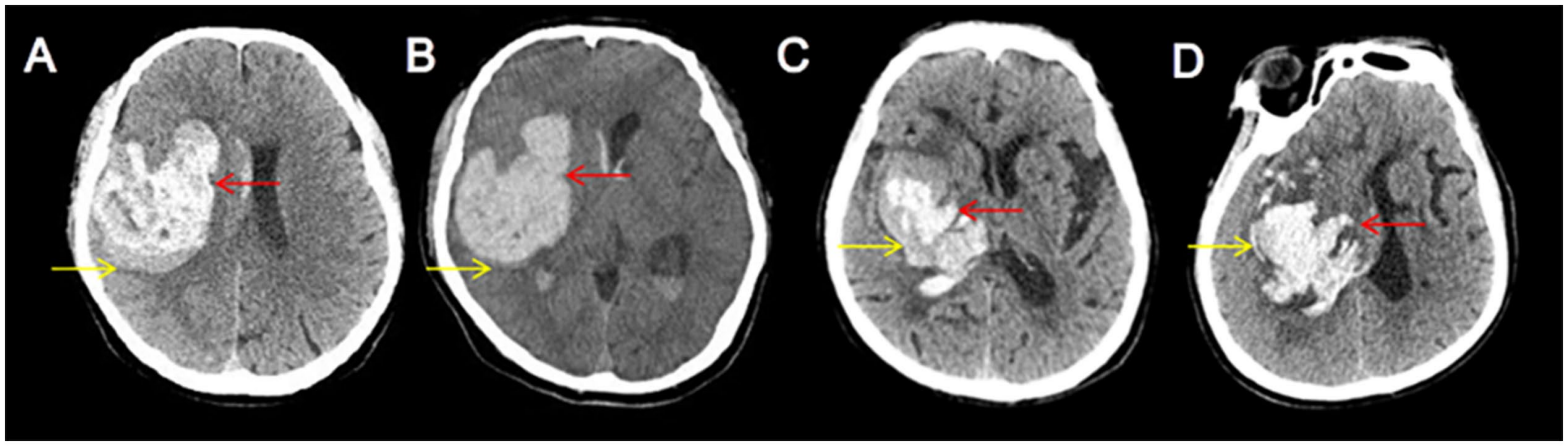
Two patients with decreased hypodensity in the blend sign. Craniocerebral CT images of two patients with ICH at admission **(A,C)** and follow-up **(B,D)**. Follow-up CT showed decreased hypodensity in the blend sign (yellow arrow). These two patients were categorized into the CHB group.

The blend sign is characterized by mixed areas of hypoattenuation and hyperattenuation with clearly defined margins. Its alterations can be dynamically monitored through repeat CT imaging. Based on the comparison of initial and follow-up CT images, the hypoattenuating area of the blend sign was categorized as unchanged (Figure 1), expanding (Figure 2), or diminishing (Figure 3). Furthermore, based on morphological changes observed in CT images of the blend sign, the patients were categorized into two groups as follows: (1) those with changes in the relatively hypoattenuating region of the blend sign (CHB group), and (2) those with no changes in the relatively hypoattenuating region of the blend sign (NHB group).

### Statistical analysis

**Figure 4:**
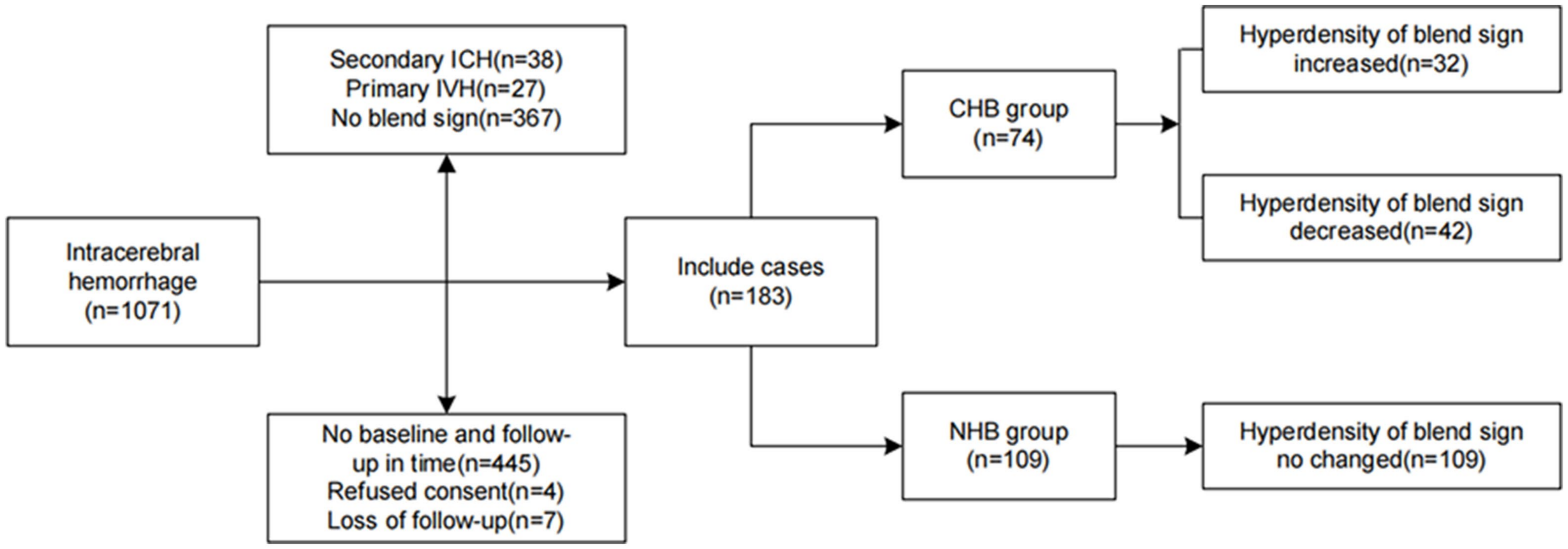
Flowchart of study patients, including those with intraventricular hemorrhage (IVH), arteriovenous malformation (AVM). The patients were categorized into the groups with and with no changes in the hypodensity area of the blend sign (CHB and NHB groups, respectively).

The statistical analysis methods employed in this study are based on our previous study (13). Univariate logistic regression analysis was utilized to identify potential independent predictors influencing changes in the blend sign appearance. The predictive accuracy of changes in the hypodensity in the blend sign for HE and adverse outcomes was examined using receiver operating characteristic curve analysis. Multicollinearity was assessed to identify the variance inflation factor (VIF) and tolerance, and collinear factors with a VIF exceeding 5 and tolerance below 0.1 were excluded to ascertain significant correlations among variables (14). Restricted cubic splines (RCS) were used to assess the non-linear relationships with the timing of baseline CT scan. The occurrence of the blend sign was determined by two investigators, whose concordance was determined using the *κ* statistic, with values of 0.21–0.4, 0.41–0.6, 0.61–0.8, and > 0.8 indicating fair, moderate, substantial, and nearly perfect agreement, respectively (Figure 4).

## Results

### Baseline characteristics

In total, the CT scans of 183 patients (145 males and 38 females) demonstrated the blend sign. The mean age of the patients was 62.1 ± 4.5 years, with a range of 60–76 years. The baseline mean hematoma volume was 28.9 ± 2.7 mL. The hematoma involved the basal ganglia in 133 patients (72.7%), cerebral lobes in 41 patients (22.4%), thalamus in 3 patients (1.6%), cerebellum in 5 patients (2.7%), and brainstem in 1 patient (0.6%). No change in the hypoattenuating region of the blend sign was observed in 109 (59.6%) patients, whereas changes were observed in 74 (40.4%) patients. In the simple factor analysis, compared to the NHB group, patients in the CHB group had a shorter interval before the baseline CT scan (*p* = 0.042), higher rate of secondary IVH (*p* = 0.01), and lower red blood cell (RBC) count (*p* = 0.013) (Table 1).

**Table 1:** Comparison of baseline characteristics between the two groups.

Characteristics	CHB group (*n* = 74)	NHB group (*n* = 109)	*Z*/*χ*^2^	*p* value
Mean age, *y* (SD)	64.5 (62.7–66.3)	63.8 (61.5–65.2)	−1.512	0.301
Gender (male, %)	56 (87.5)	89 (81.7)	0.957	0.328
History, *n* (%)				
Hypertension	57 (77.0)	75 (68.8)	1.418	0.224
Diabetes mellitus	6 (8.1)	9 (8.3)	0.001	0.971
Smoking	40 (54.1)	63 (57.8)	0.251	0.616
Alcohol consumption	30 (40.5)	60 (55.0)	3.711	0.054
History of stroke	12 (16.2)	11 (10.1)	1.505	0.220
Clinical/radiographic status at admission				
SBP	171.5 (154.7–190.0)	170.0 (148.5–188.5)	1.266	0.531
DBP	101.0 (89.0–116.0)	101.0 (89.0–113.0)	0.162	0.922
GCS on admission (points, IQR)	12(9–13)	13(11–14)	−2.398	0.017
NIHSS on admission (points, IQR)	14 (10–18)	10 (7–14)	−3.338	0.001
Time from onset to baseline CT (h)	3.0 (2.0–6.0)	5.0 (3.0–16.0)	6.366	0.042
Time from baseline CT to follow-up CT (h)	8.0 (4.0–14.0)	8.0 (4.0–15.0)	−0.723	0.962
Time from onset to follow-up CT time (h)	16.0 (8.0–21.0)	17.0 (9.0–23.0)	−0.990	0.332
Hematoma location, *n* (%)			1.148	0.887
Basal ganglia	56 (87.5)	77 (70.6)		
Lobe	15 (23.4)	26 (23.9)		
Brainstem	0 (0.0)	1 (0.9)		
Thalamus	1 (1.6)	2 (1.8)		
Cerebellum	2 (3.1)	3 (2.8)		
Secondary IVH	21 (32.8)	13 (11.9)	7.886	0.005
Hematoma volume (mL), median (IQR)	26.0 (17.0–39.0)	23.0 (15.0–40.0)	−0.666	0.506
Laboratory testing				
WBC (×10^9^/L), median (IQR)	8.3 (6.2–10.9)	8.7 (6.7–11.2)	−1.342	0.180
Neutrophils (%), mean (SD)	73.7 (61.9–84.5)	77.5 (69.7–85.2)	−1.793	0.107
Lymphocytes (%), mean (SD)	17.6 (9.7–26.6)	14.8 (9.4–20.3)	−1.928	0.054
Monocytes (%), mean (SD)	6.5 (4.4–7.9)	6.1 (4.3–7.0)	−1.815	0.073
RBC, 10^12^/L	4.7 ± 0.7	4.9 ± 0.7	2.504	0.013
Hb, g/L	144.0 ± 17.6	147.6 ± 19.0	1.266	0.207
MCV, fL	91.6 (88.8–95.0)	90.1 (86.8–94.6)	−1.875	0.060
MCHC, pg	338.0 (332.0–343.0)	338.0 (328.5–347.0)	−1.102	0.216
PLT, (×10^9^/L), mean (SD)	194.5 (151.8–234.3)	197.0 (160.0–232.5)	−0.805	0.421
K, mmol/L	3.7 (3.5–3.9)	3.7 (3.4–4.0)	−0.604	0.546
CREA, μmol/L	70.8 (59.2–87.5)	68.1 (58.1–81.7)	−0.317	0.751
INR, median (IQR)	1.0 (0.9–1.0)	0.9 (0.9–1.0)	−0.940	0.347
APTT (s), median (IQR)	33.6 (31.3–35.9)	33.5 (30.7–36.6)	−0.053	0.957
PT (s), median (IQR)	12.8 (12.2–13.2)	12.5 (12.0–13.2)	−0.836	0.403

SBP: systolic blood pressure, DBP: diastolic blood pressure, WBC: white blood cell count, RBC: red blood cell count, Hb: hemoglobin, MCV: mean corpuscular volume, MCHC: mean corpuscular hemoglobin concentration, PLT: platelet count, K: potassium, CREA: creatinine, PT: prothrombin time, APTT: activated partial thromboplastin time, INR: International Normalized Ratio.

### Morphological changes in the CT blend sign and time until baseline CT

**Figure 5:**
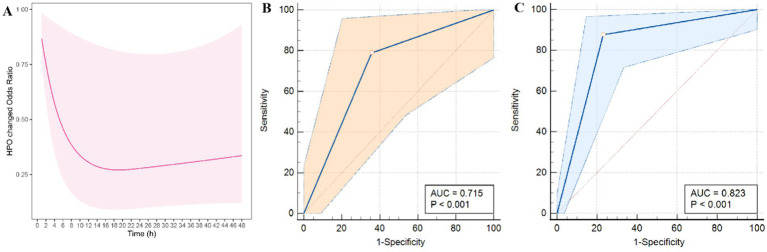
Modeled probability of significant morphological changes in the blend sign over time from symptom onset to CT imaging based on restricted cubic spline (RCS) **(A)**. Additionally, ROC curves for the prediction of HE **(B)** and poor outcomes **(C)** based on CHB are presented.

Morphological changes in the blend sign were observed in 74 (40.4%) patients. After multicollinearity analysis, RCS curve demonstrated the CHB group is associated with the time elapsed from symptom onset to CT imaging (*p* = 0.042) (Figure 5A). The median time from symptom onset to initial CT scan was 3 h for patients in the CHB group and 5 h for those in the NHB group.

### HE and prognosis in the CHB group

Univariate analysis revealed significant differences in the predictors of HE, including systolic blood pressure at admission (*p* = 0.025), secondary IVH (*p* = 0.013), CHB (*p* < 0.001), neutrophil count (*p* = 0.036), and lymphocyte count (*p* = 0.014), between the groups (Table 2). Moreover, multivariate logistic regression analysis revealed that the CHB group was an independent predictor of HE (odds ratio [OR] = 19.401, 95% confidence interval (CI) = 7.217–52.163, *p* < 0.001) (Table 3). The area under the receiver operating characteristic curve (AUC) for the inflammatory score was 0.823 (95% CI = 0.755–0.891, *p* < 0.001), suggesting that the change in the hypodensity of the blend sign is a reliable marker of HE. Similarly, univariate analysis indicated significant differences in mean age (*p* = 0.008), secondary IVH (*p* = 0.032), and changes in the hypodensity of the blend sign (*p* = 0.003) in terms of the prediction of a poor 3-month mRS score (Table 2). Subsequently, multivariate logistic regression analysis revealed that both mean age (OR = 1.017, 95% CI = 1.008–1.137, *p* = 0.027) and CHB group (OR = 2.638, 95% CI = 1.391–5.003, *p* = 0.003) were independently associated with a poor 3-month mRS score (Table 2).

**Table 2:** Univariate and multivariate analyses of potential predictors of hematoma expansion, 30-day mortality, and poor mRS score at 3 months.

	Hematoma expansion			30-day mortality			Poor 3-month mRS score (4–6)		
Characteristics	OR	95% CI	*p* value	OR	95% CI	*p* value	OR	95% CI	*p* value
Age	1.016	0.990–1.042	0.226	1.095	1.023–1.172	0.009	1.066	1.017–1.118	0.008
Gender	1.478	0.626–3.492	0.373	1.615	0.181–14.395	0.667	1.240	0.302–5.091	0.765
History									
Hypertension	0.680	0.316–1.464	0.324	0.255	0.029–2.201	0.214	0.639	0.199–2.049	0.451
Diabetes mellitus	0.994	0.301–3.281	0.992	0.467	0.046–4.782	0.521	1.469	0.154–14.038	0.738
Smoking	0.953	0.492–1.846	0.887	0.520	0.097–2.782	0.445	0.348	0.102–1.189	0.092
Alcohol consumption	1.264	0.656–2.439	0.484	0.939	0.216–4.086	0.934	0.804	0.281–2.296	0.683
History of stroke	0.646	0.255–1.635	0.356	0.368	0.062–2.190	0.272	0.681	0.152–3.044	0.615
Clinical/radiographic status at admission									
SBP	1.013	1.002–1.024	0.025	1.009	0.988–1.031	0.396	1.010	0.994–1.027	0.205
DBP	1.011	0.005–1.027	0.192	0.996	0.961–1.032	0.819	0.996	0.971–1.021	0.734
GCS on admission	1.442	0.924–2.251	0.107	1.091	0.675–0.981	0.030	3.875	0.689–0.943	0.007
NIHSS on admission	1.062	1.002–1.126	0.042	1.096	1.014–1.184	0.039	1.142	1.064–1.227	0.000
Time from onset to baseline CT	0.974	0.945–1.004	0.088	0.968	0.896–1.046	0.407	0.768	0.602–0.979	0.330
Time from baseline CT to follow-up CT	0.977	0.938–1.017	0.253	0.993	0.940–1.049	0.796	0.952	0.907–0.990	0.480
Time from onset to follow-up CT time	0.979	0.954–1.005	0.117	0.982	0.945–1.020	0.338	0.947	0.939–0.986	0.330
Hematoma location									
Basal ganglia	0.646	0.653–6.380	0.708	1.067	0.674–3.810	0.115	1.537	0.516–2.323	0.201
Lobe	0.687	0.581–8.145	0.766	1.029	0.280–5.935	0.206	1.425	0.439–3.449	0.191
Brainstem	0.766	0.197–9.398	0.676	3.215	1.251–8.794	0.016	2.238	0.224–1.598	0.002
Thalamus	0.500	0.225–6.348	0.497	0.734	0.937–5.432	0.769	0.750	0.387–1.492	0.851
Cerebellum	0.375	0.188–5.369	0.462	0.612	0.224–1.598	0.305	0.938	0.394–2.213	0.931
Hematoma volume	1.032	1.003–1.061	0.030	1.020	1.014–1.026	0.037	1.057	1.013–1.117	0.006
Secondary IVH	2.873	1.334–6.187	0.000	0.918	0.168–5.035	0.922	3.616	1.538–8.501	0.003
CHB group	21.783	8.532–55.619	0.000	0.259	0.049–1.384	0.114	3.085	1.658–5.741	0.000
Hematoma volume	1.001	0.985–1.018	0.897	1.005	0.963–1.050	0.809	1.007	0.976–1.039	0.661
Laboratory testing									
WBC	0.936	0.853–1.026	0.159	0.932	0.731–1.187	0.567	1.001	0.858–1.169	0.985
Neutrophils (%)	0.895	0.889–0.993	0.036	1.010	0.950–1.072	0.757	0.960	0.920–1.003	0.065
Lymphocytes (%)	1.639	1.104–2.433	0.014	0.978	0.903–1.060	0.591	1.038	0.985–1.095	0.164
Monocytes (%)	2.541	0.720–8.963	0.147	1.125	0.893–1.418	0.316	1.210	0.996–1.469	0.550
RBC	0.812	0.506–1.301	0.386	0.330	0.106–1.026	0.056	1.063	0.497–2.276	0.874
Hb	1.001	0.983–1.019	0.916	0.982	0.949–1.016	0.284	1.010	0.983–1.037	0.481
MCV	1.035	0.987–1.085	0.153	1.137	1.003–1.288	0.044	1.060	0.976–1.152	0.166
MCHC	1.016	0.990–1.044	0.227	0.993	0.939–1.050	0.803	1.117	0.894–1.396	0.331
PLT	1.000	0.995–1.006	0.901	0.994	0.981–1.007	0.376	0.992	0.982–1.002	0.098
K	0.584	0.279–1.220	0.152	1.673	0.471–5.944	0.426	0.972	0.359–2.627	0.955
CREA	1.000	0.995–1.004	0.842	1.003	0.988–1.018	0.693	1.001	0.989–1.013	0.847
INR	1.471	0.088–24.637	0.778	0.121	0.010–17.240	0.596	0.133	0.002–20.365	0.432
APTT	0.980	0.991–1.054	0.586	1.014	0.971–1.058	0.535	1.002	0.973–1.033	0.871
PT	1.083	0.827–1.418	0.562	0.777	0.347–1.740	0.539	0.950	0.588–1.536	0.834
Multivariate analysis									
Age	–	–	–	1.082	1.006–1.162	0.033	1.071	1.008–1.137	0.027
SBP	1.011	0.996–1.026	0.156	–	–	–	–	–	–
GCS on admission	–	–	–	0.916	0.652–1.287	0.614	1.550	0.942–2.549	0.084
NIHSS on admission	1.097	0.931–1.294	0.269	1.063	0.965–1.170	0.219	1.206	0.983–1.479	0.072
Brainstem	–	–	–	1.517	1.224–1.476	0.016	1.172	1.165–1.322	0.029
Hematoma volume	1.011	0.966–1.057	0.651	1.051	1.014–1.089	0.034	1.067	1.028–1.105	0.030
Secondary IVH	1.673	1.116–4.109	0.027	–	–	–	2.932	1.210–7.106	0.017
CHB group	19.401	7.217–52.163	0.000	–	–	–	2.638	1.391–5.003	0.003
Neutrophils (%)	1.040	0.909–1.190	0.571	–	–		–	–	–
Lymphocytes (%)	1.112	0.934–1.325	0.231	–	–	–	–	–	–
MCV, fL	–	–	–	1.090	0.949–1.251	0.223	–	–	–

**Table 3:** Sensitivity, specificity, positive predictive value (PPV), negative predictive value (NPV), and accuracy of HBC in predicting hematoma expansion (HE) and poor outcomes.

	Sensitivity	Specificity	PPV	NPV	Accuracy
HE	87.8	76.9	79.1	86.3	82.3
Poor 3-month mRS (4–6)	79.0	64.2	68.8	75.3	71.5

### Sensitivity analyses

We evaluated the diagnostic accuracy of the CHB group for predicting HE and adverse outcomes (Figure 5). The AUC values for predicting HE and 3-month unfavorable mRS outcomes were 0.823 and 0.715, respectively. The sensitivity, specificity, positive predictive value, and negative predictive value of CHB for predicting HE were 0.877, 0.768, 0.791, and 0.862, respectively, whereas these values for predicting poor outcomes were 0.789, 0.641, 0.688, and 0.752, respectively (Table 3).

## Discussion

Our present study showed that the morphology of the blend sign is variable. Comparison of the initial and follow-up CT scans provides crucial information regarding hematoma stability. Persistent hypodensity within the blend sign indicates relative stability, whereas changes in the hypodensity suggests hematoma instability, signifying increased risks of HE and an unfavorable prognosis among patients with ICH.

HE is a pivotal determinant of early neurological deterioration and subsequent fatal prognosis. A study observed that an increase in hematoma volume by 3 mL can triple the risk of death or disability in patients (15). Consequently, prompt measures to mitigate the risk of HE are imperative during the initial phase following ICH. However, the use of interventions to reduce HE in randomized clinical trials has not yielded a definitive therapy with marked impact on bleeding extent and functional outcome. Previous studies have suggested that non-contrast CT markers independently predicts the risk of HE (16). The blend sign demonstrates a notably high specificity in predicting the occurrence of HE when compared to other non-contrast CT markers (17). In line with a meta-analysis showed that the pooled sensitivity and specificity of the blend sign for predicting HE are 0.28 and 0.92, respectively (18–20). However, in the present study, we found that changes in the hypodensity in the blend sign exhibited higher sensitivity for HE, suggesting that it is a more important indicator of HE than the presence of blend sign alone. Therefore, this study also compensates for the limited predictive power of blend sign due to its low sensitivity. In previous studies, the presence of blend sign was extensively investigated (4). Our previous research demonstrated a robust association between the presence of the blend sign on the initial CT scan and the likelihood of re-hemorrhage following stereotactic minimally invasive surgery in patients with ICH (11). Despite this, few studies have explored the morphological alterations associated with the blend sign. In fact, blend sign is a time-sensitive parameter that typically undergoes dynamic changes within the initial hours following symptom onset. In the present study, changes were demonstrated in the hypoattenuating region of the blend sign in 40.4% of cases. Additionally, the RCS curve suggested that a more rapid completion of the CT scan increases the probability of detecting morphological changes in the blend sign, potentially due to the reduced CT review time or early persistent bleeding (4). A recent study demonstrated that the presence of spot sign on CT angiography is significantly associated with the time elapsed from ICH onset (21). A multicenter study involving 1,488 patients proposed an innovative concept of time-adjusted frequency of imaging markers (17), suggesting that shorter CT scan times and presence of imaging markers such as the blend sign facilitates the early detection of HE and facilitates appropriate clinical decision-making. In a study involving 1,186 patients with ICH, the presence of the blend sign was associated with a 2.514-fold higher risk of poor prognosis compared to patients without such symptoms. Furthermore, among 518 patients exhibiting the blend sign, 64.1% experienced a poor prognosis, whereas only 45.6% of the remaining 668 patients without mixed symptoms had unfavorable outcomes (22). Overall, our results are in line with those of previous studies. Previous studies have shown that advanced age is associated with more severe neurological impairment and worse long-term prognosis in patients with ICH (23). In this study, we also observed a positive correlation between age and poor outcomes in patients with ICH. Older individuals with this condition exhibited a higher mortality rate and poorer prognosis, potentially attributed to their diminished physiological reserve, impaired recovery ability, prolonged hospitalization, and increased risk of complications.

The pathophysiology of the CT blend sign remains unclear. However, it likely represents early hemorrhage characterized by relative hypodensities (24). Our previous research based on the attenuation of whole blood and blood fractions s suggests that the blend sign consists of two blood components of varying ages (8). Moreover, our study revealed a significantly elevated rate of secondary IVH in patients exhibiting changes in the hypodensity associated with the blend sign compared to those without changes. Numerous studies have demonstrated that the rate of development of ultra-early ventricular hemorrhage is closely associated with early neurological impairment (25). A study involving 160 patients with ICH found that HE occurs within the first hour after symptom onset in most cases. Therefore, CT scans conducted earlier were more likely to detect HE (7). Additionally, we found a correlation between IVH and HE in patients with ICH. Another prospective study discovered that ultra-early ventricular hemorrhage often leads to neurological deterioration (26). Furthermore, the density of the hypodense region of the blend sign indicates the cerebrospinal fluid composition. A clinical case report demonstrated that a patient with ICH rapidly developed consciousness disturbance, and subsequent CT reexamination revealed an enlargement of the hypodensity area surrounding the hematoma without any observed hematoma growth. During craniotomy for hematoma removal, an aspirable soft clot was discovered above the wall of the cerebral ventricle at the base of the hematoma cavity, which had led to the obstruction of cerebrospinal fluid circulation and exacerbated cerebral herniation in the patient. Following removal of the soft clot, midline displacement improved and neurological impairment resolved (27). Based on these findings, we propose that the development of the blend sign may be associated with cerebrospinal fluid circulation pathways. Specifically, when the hematoma is located near the ventricle and/or subarachnoid space, it is possible that the blend sign reflects an accumulation of cerebrospinal fluid in the hematoma space rather than active bleeding. Furthermore, subsequent CT scans performed at different intervals may reveal underlying changes in the hypodensity area of the blend sign.

As the predominant cellular constituent of hematoma, damaged red blood cells (RBC) undergo dissolution and subsequently release hemoglobin (Hb) within a few days following cerebral hemorrhage. The presence of Hb, along with its dissolved byproducts heme and iron, contributes to oxidative damage at the cellular level, disruption of the blood–brain barrier, neuronal demise, and irreversible brain injury (28). The CT attenuation of blood is predominantly determined by the RBC fraction. Previous studies have revealed that injecting intact RBCs into the cranial region of rats did not lead to encephaledema within 24 h; however, the introduction of lysed RBCs resulted in significant brain edema in the same time window, possibly mediated by hemoglobin (29). In our cohort study, the presence of RBCs was higher in the NHB group than in the CHB group, suggesting a correlation between lower RBC counts and hypodensity in the blend sign. Furthermore, these findings indicate the involvement of RBCs in the appearance of the blend sign. The hypodense areas in the blend sign often exhibit density values of approximately 18 HUs lower than those of the remaining parts of the hematoma. A clinical study investigating the correlation between CT values and HE revealed a higher likelihood of HE with a lower HU value of the hematoma. Considering that the HU values on CT scans with ICH primarily depend on RBC density, an elevation in the HU values of the hematoma may indicate clot contraction (30). Additionally, the inflammatory cascade resulting from RBC cleavage can induce disruption of the blood–brain barrier, leading to transcriptional and posttranscriptional alterations in ion channels and transporters within brain capillary endothelial cells. These modifications contribute to aberrant ion transportation and abnormal osmotic pressure. Furthermore, a substantial quantity of proteins infiltrate into the surrounding brain tissue space from the hematoma cavity, elevating osmotic pressure and causing water seepage from the bloodstream into the brain parenchyma, ultimately resulting in interstitial cerebral edema (31). In a study conducted on animals with ICH, it was observed that the area of “translucent” white matter adjacent to the hematoma exhibited over 10% higher water content compared with the contralateral white matter, within 1 h of the occurrence of ICH (32). Consequently, morphological changes observed in the blend sign may indicate clot contraction within the hematoma as well as alterations in intercellular fluid.

In our study, we introduced a novel term, CHB, to characterize the alterations of the hypoattenuated region within the blend sign in patients with ICH. In this study, we observed the alterations in the hypoattenuated region within the blend sign when patients were scanned earlier. In addition, we confirmed a significant correlation between the alterations in the hypoattenuated region within the blend sign and both HE and poor 3-month outcomes. Furthermore, we developed an algorithm for the identification of individuals with a heightened risk of CHB. This will assist clinicians in identifying patients who require close monitoring.

This study had several limitations. The sample size in this study was constrained, and the study only included patients with the blend sign, potentially leading to selection bias. Also, we focused solely on the morphological changes in the hypodense region of the blend sign following an ICH, without evaluating the potential predictive value of hyperdense regions of the blend sign. Besides, the hypodensity within the blend sign indicates perihematomal edema, which was not assessed in our study. Finally, we did not explore the relationship between the hematoma distribution and the ventricular circulatory system. Future studies should evaluate the associations among hypodensity in the blend sign, perihematomal edema, and the ventricular circulatory system.

## Conclusion

Changes in the relatively hypoattenuating region of the blend sign represent an active ongoing process even several hours after admission, which may be an effective tool for the bedside monitoring of HE and poor outcomes.

## Data Availability

The raw data supporting the conclusions of this article will be made available by the authors, without undue reservation.
